# Neuroinflammation as a Target for Intervention in Subarachnoid Hemorrhage

**DOI:** 10.3389/fneur.2018.00292

**Published:** 2018-05-02

**Authors:** Airton Leonardo de Oliveira Manoel, R. Loch Macdonald

**Affiliations:** ^1^Adult Critical Care Unit, Hospital Paulistano – United Health Group, São Paulo, Brazil; ^2^Keenan Research Center for Biomedical Science, Department of Surgery, Li Ka Shing Knowledge Institute, University of Toronto, Toronto, ON, Canada; ^3^Division of Neurosurgery, St. Michael’s Hospital, Labatt Family Centre of Excellence in Brain Injury and Trauma Research, Keenan Research Centre for Biomedical Science, Department of Surgery, Li Ka Shing Knowledge Institute, University of Toronto, Toronto, ON, Canada

**Keywords:** neuroinflammation, subarachnoid hemorrhage, early brain injury, secondary brain injury, vasospasm, delayed cerebral ischemia

## Abstract

Aneurysmal subarachnoid hemorrhage (SAH) is a sub-type of hemorrhagic stroke associated with the highest rates of mortality and long-term neurological disabilities. Despite the improvement in the management of SAH patients and the reduction in case fatality in the last decades, disability and mortality remain high in this population. Brain injury can occur immediately and in the first days after SAH. This early brain injury can be due to physical effects on the brain such as increased intracranial pressure, herniations, intracerebral, intraventricular hemorrhage, and hydrocephalus. After the first 3 days, angiographic cerebral vasospasm (ACV) is a common neurological complication that in severe cases can lead to delayed cerebral ischemia and cerebral infarction. Consequently, the prevention and treatment of ACV continue to be a major goal. However, most treatments for ACV are vasodilators since ACV is due to arterial vasoconstriction. Other targets also have included those directed at the underlying biochemical mechanisms of brain injury such as inflammation and either independently or as a consequence, cerebral microthrombosis, cortical spreading ischemia, blood–brain barrier breakdown, and cerebral ischemia. Unfortunately, no pharmacologic treatment directed at these processes has yet shown efficacy in SAH. Enteral nimodipine and the endovascular treatment of the culprit aneurysm, remain the only treatment options supported by evidence from randomized clinical trials to improve patients’ outcome. Currently, there is no intervention directly developed and approved to target neuroinflammation after SAH. The goal of this review is to provide an overview on anti-inflammatory drugs tested after aneurysmal SAH.

## Introduction

Aneurysmal subarachnoid hemorrhage (SAH) is a complex cerebrovascular disease with profound systemic complications (1–3). Despite the advances achieved in the management of those patients, high disability and mortality rates continue to devastate this patients’ population (4).

Aneurysm rebleeding is the most threatening early neurological complication and usually occurs in the first 24 h after the initial hemorrhage (5, 6). However, delayed cerebral ischemia (DCI) remains the major cause of morbidity and mortality among patients who survive after repair of the ruptured aneurysm (7, 8).

For decades, angiographic cerebral vasospasm (ACV), a common neuroradiological finding after SAH, has been proposed as the primary cause of DCI and DCI-related cerebral infarction (9). In turn, severe angiographic vasospasm can cause DCI and DCI-related cerebral infarction, which made the prevention and treatment of ACV a major target in the management of SAH. Crowley et al. analyzed 381 patients who were part of a randomized clinical trial and all of whom underwent serial CT scanning and angiography (10). Angiographic vasospasm was graded as none/mild (209, 55%), moderate (118, 31%), or severe (54, 14%). Delayed cerebral infarctions occurred in 3% of those with no/mild, 10% with moderate, and 46% with severe vasospasm. The data also show that a minority of patients can develop DCI without ACV (10). Angiographic vasospasm occurs in approximately 70% of patients during the first 2 weeks after SAH, but the incidence of DCI is only around 30% (7). Some patients with severe ACV do not develop ischemia or infarction for the same reasons that a patient with a carotid artery occlusion may not, which includes (a) the recruitment of collateral blood flow through the anterior and posterior communicating arteries (i.e., the primary collateral pathway), or through the ophthalmic artery and leptomeningeal vessels (i.e., the secondary collateral pathway) (11, 12) and (b) the cerebral vasoreactivity status (i.e., intact versus impaired) (13). In addition, patients may develop cerebral infarction in vascular territories that are not affected by ACV (14, 15). Moreover, enteral nimodipine is the only pharmacological treatment shown to improve functional outcome, despite the fact that it does not lead to marked cerebral vasodilation (16, 17).

Also, a meta-analysis that included 14 randomized controlled trials in SAH, involving 4,235 patients, showed that the tested drugs significantly reduced angiographic vasospasm, but clinical outcomes remained unaffected (18).

Because of the failure of several pharmacological trials and the dissociation between vasospasm-related morbidity and functional outcomes, other possible mechanisms, such as early brain injury, cortical spreading ischemia, microthrombosis, cerebral autoregulation impairment, and capillary transit time heterogeneity, are thought to play a role in the pathophysiology of DCI and DCI-related cerebral infarction (1, 3, 19, 20). These additional mechanisms tend to be more difficult to assess clinically and the dissociation mentioned above could also be due to drug toxicity and off-target effects, effects of rescue therapy, inadequate trial sample size and other trial design problems, and testing of only one dose and dose regimen.

Another common biochemical mechanism after brain injury is neuroinflammation, which may also contribute to many of the causes of brain damage after SAH. Subarachnoid blood and subsequent hemoglobin degradation can in turn trigger the inflammatory cascade, which also develops in the brain probably secondary to ischemia, blood–brain barrier breakdown, and such. There is an increasing interest in the understanding of the role of neuroinflammation in the pathophysiology of DCI and other severe complications after SAH.

The main goal of this review article is to summarize the concept of neuroinflammation following SAH, and its potential contribution to ACV, DCI, and systemic complications. We also address the possible benefits of targeting the inflammatory cascade after aneurysmal SAH. Finally, we review studies of drugs directed at neuroinflammation after SAH (Table 1).

**Table 1:** Summary of medication with anti-inflammatory activity tested in SAH.

Drug	Design	Dose	Patients	Outcome	Mechanism of action	Conclusion
Cyclosporin A (21)	Prospective cohort study	Loading dose of 7.5 mg/kg Maintenance: enteral administration every 12 h for two doses, to maintain levels of 50–400 ng/kg	Nine patients with Fisher Grade 3	GOS at 6 months	Prevent vasospasm, inhibit IL-2 production, and prevent T-cell dysfunction	CycA proved safe to use but failed to prevent the development of cerebral vasospasm or delayed ischemic deficits in patients considered at high risk

Cyclosporin A (22)	Randomized clinical trial	Cyclosporine A orally 6–9 mg/kg/day to maintain level of cyclosporine in the blood at 100–400 ng/ml	25 patients (9 received treatment)	*Neurological state*	Prevent vasospasm, inhibit IL-2 production, and prevent T-cell dysfunction	Patients treated with early clipping (up to 72 h after SAH) plus cyclosporine A had significantly better “neurological outcome” than controls

Methylprednisolone (23)	Case-control study	Methylprednisolone started within 3 days following the tapering regimen: – 30 mg/kg q6h × 12, 15 mg/kg q6h × 4, 7.5 mg/kg q6h × 4, 3 mg/kg q6h × 4, and 1.5 BID × 2 – 30 mg/kg before aneurysm operation	42 patients (21 received treatment)		Corticosteroids have multiple anti-inflammatory actions, mostly on chronic inflammation	

Methylprednisolone (24)	Double-blind, placebo-controlled, randomized trial	Methylprednisolone 16 mg/kg IV every day for 3 days (started within 6 h after angiographic diagnosis of aneurysm rupture), or placebo	95 patients	*Symptomatic vasospasm*^a^ mRS in living patients GOS at 1 year after SAH, in all patients	Corticosteroids have multiple anti-inflammatory actions, mostly on chronic inflammation	The treatment did not reduce the incidence of *symptomatic vasospasm* but improved functional outcome

Hydrocortisone (25)	Double-blind, placebo-controlled, randomized trial	Hydrocortisone 3 g IV BID, repeated 6 times	140 patients, 71 patients who received hydrocortisone	Mental, speech, and motor function	Hydrocortisone reduces vascular sensitivity to various vasoconstrictive stimuli. It inhibits phospholipase to reduce production of prostaglandins. It stabilizes the cell membrane and prevents cerebral edema	Patients who received hydrocortisone showed improvement in mental, speech, and motor function

Dexamethasone (26)	A propensity score analysis	Dexamethasone 4 mg q6h, then tapering down by 1 mg per dose every 24 h until discontinuation	309 patients, 101 (33%) received treatment	Unfavorable outcome (mRS > 3)		Dexamethasone was associated with a significant reduction in mRS >3, but its use had no association with DCI or infection

Simvastatin (1, 27)	Meta-analysis	Simvastatin 40 or 80 mg/day up to 21 days	Six randomized clinical trials, including 1,053 patients	Delayed ischemic deficit and delayed cerebral infarction	Neuroprotection independent of cholesterol reduction and exclusively associated with upregulation of endothelial nitric oxide synthase	No effect on delayed ischemic deficit, delayed cerebral infarction, mRS ≤2, vasospasm, ICU stay, hospital stay, and mortality

Acetylsalicylic acid (aspirin), ADP P2Y_12_ receptor antagonists (thienopyridines), and thromboxane synthase inhibitors (28)	Meta-analysis	Multiple regimens^b^	Seven randomized clinical trials, including 1,385 patients	Poor outcome (death, or dependence on help for activities of daily living)	Aspirin exerts its antiplatelet activity by the irreversible inhibition of COX-1 enzyme, thereby blocking the formation of thromboxane A2 in the platelets Because aspirin block the COX-1 enzyme, which decreases prostaglandin synthesis, leading to an anti-inflammatory effect Thienopyridines are ADP P2Y_12_ receptor antagonists (e.g., ticlopidine) that inhibit the intracellular pathways leading to platelet activation	No effect on case fatality, aneurysmal rebleeding, poor outcome, secondary brain ischemia, and intracranial hemorrhagic complications. Ticlopidine was the sole antiplatelet agents associated with a significant reduction in the occurrences of a poor outcome (RR 0.37, 95% CI 95% CI 0.14–0.98), however, this result was based on one small RCT

Non-steroidal anti-inflammatory (29)	A propensity score-matched study	Multiple regimens not described^c^	178 patients were matched [89 received non-steroidal anti-inflammatory drug (NSAIDs), 89 did not]	Clinical outcomes included 6-week mortality, 12-week modified Rankin scale (mRS) score, DCI, and delayed ischemic neurological deficit (DIND)	NSAIDs inhibit COX, which decreases prostaglandin synthesis; ibuprofen inhibits expression of endothelial adhesion molecules and reduces subarachnoid inflammation	No significant difference in functional outcome, in the development of DINDs, angiographic vasospasm, or need for rescue therapy

Clazosentan (19)	Meta-analysis	Multiple regimens^d^	Four randomized clinical trials, including a total of 2,181 patients	Glasgow Outcome Scale—extended and mortality	Synthetic endothelin A receptor antagonist, with reduction of angiographic vasospasm	Clazosentan had a significant impact in the reduction of DINDs and delayed cerebral infarction. However, functional outcomes or mortality were unaffected Side effects, such as hypotension, anemia, and pulmonary complications may have reduced the beneficial effects of the drug

Cilostazol (30)	Randomized, single-blind study		109 patients undergoing clipping of ruptured aneurysms		Selective phosphodiesterase III inhibitor, which inhibits platelets through an increase in intraplatelet cAMP levels. It has an antithrombotic, vasodilatory, anti-smooth muscle proliferation, and cardiac inotropic and chronotropic effects. Cilostazol also exhibits anti-inflammatory properties including inhibiting microglial activation	A multicenter randomized clinical trial of cilostazol has shown a decrease in angiographic vasospasm but no improvement in outcomes 6 months after SAH. Cilostazol significantly reduced angiographic vasospasm, DCI and cerebral infarction but had no effect on outcome

Interleukin-1 receptor antagonist (IL-1Ra) (31)	A small Phase II, double-blind, randomized controlled study	IL-1Ra (500 mg bolus, then a 10 mg/kg/h infusion for 24 h)	13 patients, 6 patients received IL-1Ra	Primary outcome: changein CSF IL-6 between 6 and 24 h	IL-1Ra limits brain injury in experimental stroke and reduces plasma inflammatory mediators associated with poor outcome	IL-1Ra appears safe in SAH patients. The concentration of IL-6 was lowered to the degree expected, in both CSF and plasma for patients treated with IL-1Ra. This did not reach statistical significance

Dual antiplatelet therapy (aspirin + clopidogrel) (32)	Single center retrospective study	Not described	161 patients (85 patients received)	Frequency of symptomatic clinical vasospasm and DCI and of hemorrhagic complications	Aspirin has an anti-inflammatory and antiplatelet effect thorough the blockade of COX-1 enzyme Clopidogrel is an antiplatelet agent (ADP P2Y_12_ receptor antagonists)	The use of DAPT was associated with a lower risk of clinical vasospasm and DCI in patients treated for SAH, without an increased risk of hemorrhagic complications

Albumin (33)	Open-label, dose-escalation, Phase I pilot study	Tier 1 = 0.625 g/kg Tier 2 = 1.25 g/kg Tier 3 = 1.875 g/kg Tier 4 = 2.5 g/kg The treatment was used for 7 days	47 patients received treatment 20 in Tier 1, 20 in Tier 2, and 7 in Tier 3	This was a dose-escalation study; therefore, the maximum tolerated dose of albumin was established. Tolerability was based on the rate of severe-to-life-threatening heart failure and anaphylactic reaction. Also, functional outcome at 3 months was assessed	Antioxidant and scavenger properties Modulate apoptosis Enhance microcirculatory blood flow Increase organ blood flow Decrease leukocyte rolling and adherence, and reduce the inflammatory response	Doses up to 1.25 g/kg/day × 7 days were well tolerated. Functional outcome trended toward better responses in those subjects enrolled in Tier 2 compared with Tier 1 (OR, 3.0513; CI, 0.6586–14.1367)

*^a^Symptomatic vasospasm (DINDs associated with angiographic arterial narrowing or accelerated flow on TCD, or both)*.

*^b^Suppositories of 100 mg ASA for 21 days after surgery; ASA 300 mg twice daily orally or rectal retention enema, starting 72 h after admission, before surgery; ticlopidine 100 mg 3 times a day orally for 2 weeks after the hemorrhage; dipyridamole 100 mg/day orally or 10 mg/day intravenously starting immediately after admission; first group 80 mg OKY-046 per day, second group 400 mg OKY-046 per day, by continuous infusion until 10–14 days, starting immediately after surgery; cataclot 1 μg/kg/min via continuous infusion, starting after surgery continued for 8–14 days; Suppositories with 100 mg ASA once daily, starting after surgery for 14 days*.

*^c^Salicylates (aspirin), propionic acid derivatives (ibuprofen, naproxen), acetic acid derivatives (indomethacin, ketorolac, diclofenac), enolic acid derivatives (meloxicam), and selective cyclooxygenase-2 inhibitors (-coxib’s)*.

*^d^GOS, Glasgow outcome score; mRS, modified Rankin scale; DCI, delayed cerebral ischemia; COX, cyclooxygenase; SAH, subarachnoid hemorrhage; CSF, cerebral spinal fluid*.

## Search Strategy

A PubMed search was performed from inception to November 2017 for articles published in English by using the terms “Subarachnoid Hemorrhage” [Mesh] AND (“Neuroinflammation” [Title/Abstract] OR “inflammation” [Title/Abstract]). A total of 272 articles were found, including 155 articles on human subjects. The authors’ own databases additionally were used as a source for this review article. This review was limited to studies in humans, because the literature on experimental animal models is vast, and it is beyond the scope of this article.

### DCI and Angiographic Vasospasm

The majority of patients (approximately 70%) develop some radiological degree of vasospasm between 3 and 14 days after a single SAH (9). However, only 30% of those will progress to develop DCI and even fewer to delayed cerebral infarction (15, 34). While ACV is an important contributor to DCI, there is evidence that other processes are involved (1, 3, 35–38).

Two major neurological consequences of aneurysmal SAH are currently described, early brain injury and ACV/DCI. Early brain injury refers to the acute effects of aneurysm rupture. Arterialized blood flows into the subarachnoid space, which suddenly increases the intracranial pressure. Consequently, the cerebral perfusion pressure is acutely reduced leading to a reduction in cerebral blood flow that produces global cerebral ischemia. Clinically, the phenomenon is characterized by transient loss of consciousness that can progress to intracranial circulatory arrest in severe cases (39, 40). There also are probably effects from the subarachnoid blood as well as brain injury from other physical and biochemical processes. Ischemia with its associated cell death causes influx of inflammatory cells, activation of microglia and neuroinflammation. Patients who survive the initial hemorrhage are at risk of DCI that is the main determinant of unfavorable outcome after SAH (41). DCI is a clinical syndrome characterized by “the occurrence of focal neurological impairment (such as hemiparesis, aphasia, apraxia, hemianopia, or neglect) and/or a decrease of at least two points on the Glasgow coma scale (either on the total score or on one of its individual components, such as eye, motor on either side, or verbal) (7). This should last for at least 1 h, is not apparent immediately after aneurysm occlusion, and cannot be attributed to other causes by means of clinical assessment, computed tomographic, or magnetic resonance imaging of the brain and appropriate laboratory studies”. The pathophysiology of DCI is multifactorial and remains to be completely elucidated, however, it is hypothesized that ACV is one if not the primary contributor, along with cortical spreading ischemia, impaired cerebral autoregulation and microcirculation constriction, microthrombosis, capillary transit time heterogeneity, and neuroinflammation (1, 3, 35–38, 42). The risk of DCI is increased by the amount of subarachnoid blood (i.e., the volume, density, and persistence of blood in the subarachnoid space) (43–45); by the initial level of consciousness (i.e., early brain injury) (46, 47); and by factors that alter the relationship between the brain oxygen/glucose supply and demand (1).

### Inflammation as an Additional Contributor to DCI

There is recent evidence suggesting that neuroinflammation may play an important role in the damage of cerebral cells after SAH (42, 48). The products of erythrocyte degradation in the subarachnoid space lead to the accumulation of hemoglobin and its products (i.e., methemoglobin, heme, and hemin), which activate toll-like receptor 4, initiating the inflammatory cascade. Microglia, the resident inflammatory cells in the central nervous system, are activated, which triggers the upregulation of a large number of endothelial adhesion molecules, allowing the inflammatory cells to reach the subarachnoid space (49). Macrophages and neutrophils, once in the subarachnoid space, start the process of phagocytosis of the product of degraded red cells, with the attempt of removing the extravascular hemoglobin, the process of which may promote neuronal healing (50). The clearance of hemoglobin from subarachnoid space depends on and is accelerated by the ligation of hemoglobin to haptoglobin. The complex hemoglobin/haptoglobin is than phagocyted by immune cells (51).

Macrophages and neutrophils are indispensable for the clearance of subarachnoid blood, however, they may become imprisoned within the subarachnoid space after the eventual reestablishment of the blood–brain barrier, and also because of the changes occurring in the cerebral spinal fluid (CSF) flow. The main problem with the entrapment of inflammatory cell in the subarachnoid space is that they suffer degranulation, releasing several inflammatory and vasoactive factors, such as endothelins. Also, these inflammatory and vasoactive factors are not restricted to the subarachnoid space, but generalized throughout the central nervous system, inducing cerebritis, aseptic meningitis, and cerebral vasoconstriction (50).

### Monitoring Inflammation After SAH

Several methods exist to monitor systemic and neurological inflammation in the patients with SAH. They include classic systemic signs of inflammation such as fever, leukocytosis, and systemic inflammatory response (SIRS), as well as the evaluation of pro- and anti-inflammatory cytokines in the peripheral blood, CSF, and cerebral extracellular fluid (52).

Increased levels of inflammatory cytokines are found in the CSF, cerebral extracellular fluid, and blood of patients with SAH (53). Two sets of authors reviewed published data reporting biomarkers for assessing outcome after SAH (54, 55). Neuron and astrocyte proteins, cell adhesion proteins, and extracellular matrix molecules, vascular, coagulation, and cardiac proteins have all been measured in various studies of CSF and blood after SAH. Inflammatory substances in blood that have been shown to be elevated after SAH included C-reactive protein (CRP), tumor necrosis factor α (TNF-α), and interleukin-1 receptor antagonist (IL-1Ra) and IL-6 (IL-6). Elevated levels of TNF-α, IL-1Ra, IL-6, and IL-8 were also described in the CSF (56–58). IL-1Ra concentrations were measured in blood and were significantly higher after SAH than in controls, in poor-grade SAH patients compared to good grades and in patients who had unfavorable as compared to favorable outcome (56). DCI further increased the concentration of IL-1Ra.

In one study, CSF TNF-α concentrations increased between 4 and 10 days after SAH in patients with unfavorable outcome whereas they did not if there was favorable outcome (56). The same rough correlation of increased CSF TNF-α and unfavorable outcome was reported by other investigators (59). Similarly, CSF levels of IL-6 and IL-8 were shown to be significantly elevated in SAH patients compared to controls, and this elevation was associated with the development of symptomatic vasospasm between day 5 and 7 (58).

Subarachnoid increases in IL-1, IL-6, and TNF-α may precede the hemodynamic abnormalities detected by transcranial Doppler and to be associated with the development of severe ACV diagnosed by transcranial Doppler ultrasound.

Elevated IL-6 levels also may be an early marker of DCI and unfavorable outcome. Muroi et al. showed in a cohort of 138 SAH patients that early elevation of IL-6 (days 3–7) was significantly associated with the occurrence of DCI and unfavorable outcome, after the adjustment for confounding factors such as infection and therapeutic hypothermia (60). In addition, elevated IL-6 levels detected in the cerebral extracellular fluid by cerebral microdialysis in patients of all grades predicted the occurrence of DCI in one study (61).

Complement system is another important component of inflammatory response after SAH. Complement activation and the formation of membrane attack complex contribute to the development of cerebral edema and the disruption in the blood–brain barrier. Interestingly, the process of complement activation and membrane attack complex formation is also implicated in the inflammation and degradation of the cerebral aneurysm wall, with consequent aneurysm rupture (62).

The complement activation in association with the formation of membrane attack complex may contribute to the development of angiographic vasospasm (63). In one study investigated the role of sequential measurement of serum complements (i.e., CH50, C3, C4) in the first 3 weeks of hemorrhage. Hunt and Hess grade was associated with C4 levels, and C4 levels were also markedly reduced in patients with severe angiographic vasospasm with neurological deficits (64).

Although, the measurement of cytokines and their receptors in the CSF, cerebral extracellular fluid, and blood seem to be feasible, their use remains experimental. Also, inflammation is the fundamental response of tissues to injury and is generally required for tissue healing. There are situations where inflammation is detrimental. Therefore, it is going to be very complex to determine which aspect of cerebral inflammation is beneficial or harmful (52). More adequate, well controlled and thus basically prospective multicenter studies would be needed before we could draw conclusions on the clinical utility of inflammatory biomarkers (53).

### Inflammation, Brain Injury, and Systemic Complications

There is systemic immune response activation after SAH and it is commonly manifested by high levels of circulating cytokines, such as IL-1, IL-6, and TNF-α (65). These are key mediators of systemic inflammation. Clinically, this process is usually manifested by fever, leukocytosis, tachycardia, and tachypnea. These are components of SIRS, which has been defined by the presence of two or more of the following: temperature <36 or >38°C, heart rate >90 bpm, respiratory rate of >20 breaths/min, and white blood cell count of <4,000 or >12,000/mm^3^ (66, 67). In 2 reports that included 276 and 413 patients with SAH, respectively, SIRS was present on admission in over half and in 63–85% within 4 days (68–70). Its occurrence was associated with poorer neurological grade, larger amount of subarachnoid blood on computed tomography. In one of the two studies, it was an independent predictor of angiographic vasospasm, systemic complications, unfavorable outcome, and death.

Systemic inflammatory response is a relatively nonspecific clinical syndrome that reflects a complex interaction among inflammation, coagulation, sympathoadrenal activation, and endothelial cell activation and dysfunction (71). This complex process generates and perpetuates tissue hypoperfusion, ultimately culminating with microthrombosis and compromised microcirculation blood flow, which is manifested by the multi-organ dysfunction syndrome (71, 72). In addition, catecholamines released into the systemic circulation after SAH may induce cardiac and pulmonary dysfunction (i.e., myocardial stunning and neurogenic pulmonary edema) (73, 74). Sympathoadrenal activation, represented by high circulating catecholamine levels, has also been shown to be highly associated with coagulopathy, endotheliopathy, and functional outcome in patients with isolated traumatic brain injury (75, 76).

Other indirect surrogate markers of systemic inflammatory activity after SAH include hyperthermia, elevated white blood cell count, hyperglycemia, high erythrocyte sedimentation rate, high CRP, transthyretin, and negative nitrogen balance (60, 64, 66, 72–82). Those systemic inflammatory parameters are associated with angiographic vasospasm, DCI, and unfavorable outcome (66, 68).

### Platelet Activation and Inflammation After SAH

Inflammation is intimately related to platelet adhesion, activation of coagulation cascade, and consequently the formation of microthrombi (82). Injury to a blood vessel leads to endothelial platelet adhesion, which triggers the coagulation cascade. Thrombin, von Willebrand factor, and collagen are capable of activating the platelets, which release a large number of procoagulant factors, responsible for the development of a hemostatic clot (82–84).

In SAH patients, microthrombosis was described in an autopsy study of seven patients who died within 3 days of SAH (85). The density of thrombi (microclot burden) was associated with clinical and radiological evidence of delayed ischemia. Also, microclot burden was significantly associated with histological evidence of ischemia.

Additional evidence for microthromboembolism includes a prospective study of SAH patients monitored with transcranial Doppler (86). Microembolic signals were detected in 16 of 23 patients (70%), and 44 of 138 arteries (32%) monitored. Microembolic signals were more common in patients with compared with patients without clinical vasospasm (83 versus 54%). These data at least show an association between inflammation, activation of coagulation cascade and formation of microthrombi after SAH and may be an additional component of DCI (83).

Inflammation and platelet activation have been associated with both early brain injury and DCI after SAH (82, 84). Frontera et al. (82), in a prospective study enrolled 106 consecutive SAH patients, comparing them with 26 control subjects. Compared to controls, SAH patients demonstrated higher levels o inflammatory biomarkers (i.e. higher levels of C-reactive protein), and higher levels of platelets activation by thromboelastography. Platelet activation and inflammation were related to the severity of early brain injury, and it was also associated with the development of DCI and unfavorable functional outcome (82).

Interestingly, nimodipine has been shown to increase endogenous fibrinolysis, which may partially explain the benefit of nimodipine use by the reduction in microthrombosis after SAH (87). However, the exact mechanism by which nimodipine exerts its beneficial effects remains to be determined, but its probably multifactorial, including fibrinolysis, inhibition of cortical spreading ischemia, and the blockage of calcium influx to neurons after ischemia (1, 20).

### Antiplatelet Drugs After SAH

**Figure 1:**
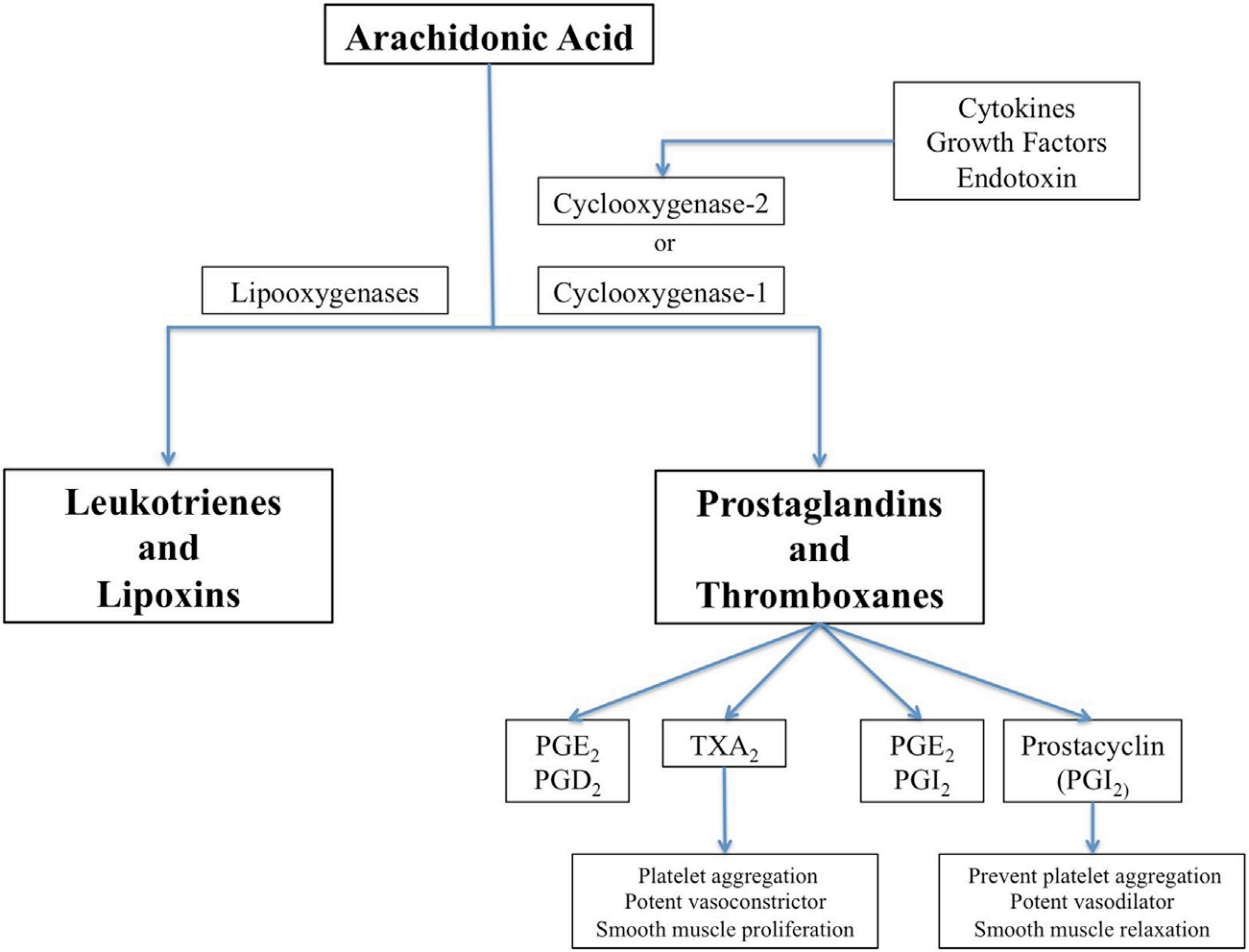
The biochemical pathway for synthesis of prostaglandins, thromboxanes, leucotrienes, and lipoxins, showing cyclooxygenase (COX)-1 which is constitutively expressed in many cells and COX-2 that is induced by inflammatory stimuli.

Cyclooxygenase (COX) enzymes are fundamental for the balance of vascular homeostasis (88). Thromboxane A2 (TxA_2_) is released by platelets as a result of COX-1 activity, and prostacyclin is synthetized mainly in vascular endothelium by COX-2 (Figure 1). TxA_2_ induces platelet aggregation, vasoconstriction, and smooth muscle proliferation, while prostacyclin antagonizes TxA_2_ effects in the macrovascular endothelium through smooth muscle relaxation and vasodilator effects. Prostacyclin also inhibits platelet aggregation through prostacyclin receptors.

**Figure 2:**
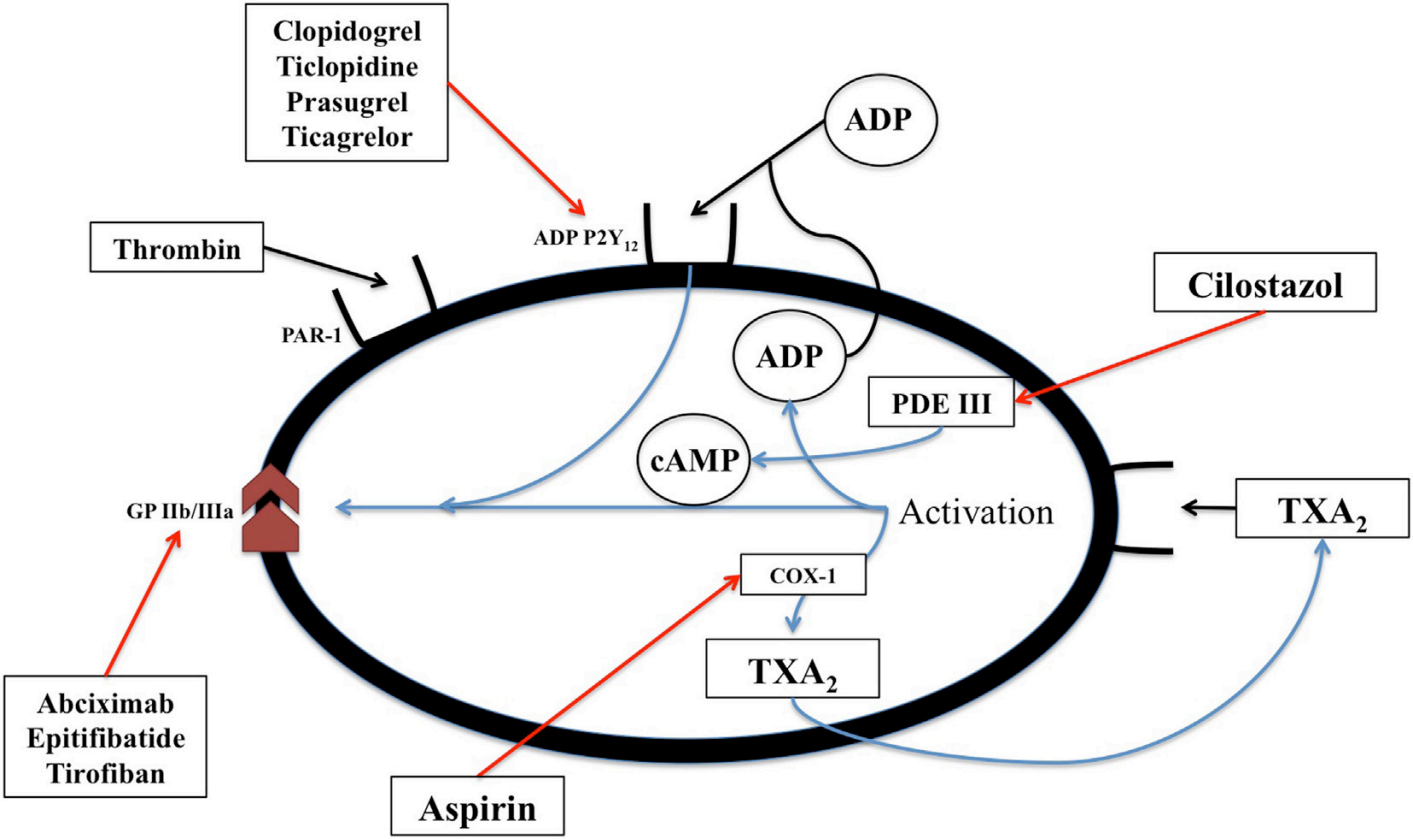
The biochemical pathways and receptors on platelets along with the site of action of non-steroidal anti-inflammatory drugs as well as some physiologic mediators of platelet degranulation and aggregation. This figure was modified from Angiolillo (89).

Acetylsalicylic acid [aspirin, a non-steroidal anti-inflammatory drug (NSAID)] inhibits cyclic prostanoid synthesis (TxA_2_, prostacyclin and other prostaglandins, Figure 2). The mechanism of its antithrombotic effect is by irreversible acetylation of COX-1, an enzyme constitutively expressed platelets and most other cells. This inhibits platelet aggregation. Its anti-inflammatory effects are due to inhibition of COX-2, which is induced by inflammation and generates inflammatory prostaglandins.

Dorhout Mees et al. conducted a meta-analysis of seven randomized clinical trials (1,385 patients) that evaluated the effect of antiplatelet drugs on outcome, case fatality, secondary ischemia, hemorrhagic intracranial complications, and aneurysm rebleeding in patients with SAH (28). The studies tested different drugs and regimens, including suppositories or oral aspirin, oral ticlopidine, oral or intravenous dipyridamole, continuous infusion of OKY-046, and continuous infusion of cataclot (90–94). There was no effect of the antiplatelet drugs on any of the outcome measures. Ticlopidine was associated with a significant reduction in poor outcome but this was based on one randomized trial of 135 patients (95).

### Steroids for SAH

Corticosteroids are anti-inflammatory. They inhibit the formation of adhesion molecules, arachidonic acid metabolites, cytokines, and chemokines. The mechanism of action that accounts for their anti-inflammatory effects is direct binding of the glucocorticoid to the glucocorticoid receptor, forming a glucocorticoid/glucocorticoid receptor complex. This is transported in to the nucleus where it binds to glucocorticoid responsive elements in the promoter region of genes, or interacts with other transcription factors, in particular activating protein-1 or nuclear factor-κB (96).

Several different types doses and dose regimens of corticosteroids have been studied in patients with SAH. Overall, the studies show mixed results and sample sizes are too small to draw conclusions among other shortcomings of the studies (23–25). The largest study was a retrospective cohort that included 309 patients, 101 (33%) who received dexamethasone. Dexamethasone significantly reduced in the odds of unfavorable outcome although it had no effect on DCI or the rate of infections (26). Glucocorticoids are not recommended for routine use in patients with SAH.

Important to mention is that a large randomized clinical trial, including more than 10,000 patients with moderate-to-severe traumatic brain injury, compared the effect of 48 h infusion of methylprednisolone with placebo. The risk of death from all causes was increased in the intervention group (97, 98). The exact cause of worse outcome with the use of steroids after traumatic brain injury is not well established.

### Immune-Suppressive Treatment in SAH

There are at least two studies that tested the use of immune-suppressive treatment in SAH. The first study was a randomized clinical trial, which included 25 patients (22). Nine received the intervention (cyclosporine A orally 6–9 mg/kg/day to maintain level of cyclosporine in the blood at 100–400 ng/ml). Patients treated with early clipping (up to 72 h after SAH) plus cyclosporine A had significantly better neurological outcome. The second study treated nine SAH patients with Fisher Grade 3 SAH with cyclosporine A (21). Despite the small number of patients, cyclosporine A was considered to be safe but to not prevent ACV or DCI.

Many SAH patients admitted to the critical care unit are exposed to invasive procedures and catheter placement, such as mechanical ventilation, external ventricular drain, invasive intracranial monitors, and vascular catheters. These invasive tools are fundamental for the management of poor-grade SAH patients (1), however, they may increase the risk of hospital-acquired infections (99). Therefore, the use of immunosuppressive treatment, including the use of glucocorticoids, might increase the risk of infection in this patient population, which increases the hospital morbidity and mortality.

### NSAIDs for SAH

Non-steroidal anti-inflammatory drugs inhibit COX (88). Traditionally they inhibited COX-1 and COX-2 although there are some specific COX-2 inhibitors. Thus, they inhibit inflammation mediated by COX-2. While aspirin is the classic NSAID, the effects differ between the newer NSAIDs and from aspirin probably due to the relative potencies against COX-1 and COX-2 and their effects on other targets. For example, ibuprofen reduces the expression of adhesion molecules by the endothelium, which in turn decreases the inflammation in the subarachnoid space. In a propensity score-matched study, Nassiri et al. studied 178 patients with SAH who were matched by propensity scoring into 89 who received NSAIDS and 89 who did not (29). Use of NSAIDs was associated with lower in-hospital mortality and shorter intensive care and hospital stay. There was, however, no significant difference in functional outcome, in the development of delayed ischemic neurological deficits, angiographic vasospasm or need for rescue therapy. Muroi and colleagues found that NSAID use was associated with lower systemic IL-6 and CRP concentrations and with better outcome in 138 patients with SAH (100). There is at least one randomized clinical trial of NSAIDs in 81 patients with SAH (101). Meloxicam had no significant effect on ACV or clinical outcome compared to placebo. Currently, the treatment of SAH patients with NSAIDs cannot be recommended.

### Other Potentially Anti-Inflammatory Drugs

Several other medications with anti-inflammatory activity have been tested in patients with SAH (Table 1). The statins are the best studied and have been completely ineffective at least in the doses and dose regimens studied (102). Albumin has multiple systemic and cerebral effects, including antioxidant and scavenger properties; the capacity to modulate cellular apoptosis; enhance of microcirculatory and organ blood flow; and an anti-inflammatory effect through the decrease in leukocyte rolling and adherence. Albumin has been studied in a dose-escalation, Phase I, pilot study (33). Doses up to 1.25 g/kg/day × 7 days were well tolerated, with a trend toward better 3-month functional outcome. A Phase III placebo control trial will be carried out in the near future.

## Conclusion

There is an increasing interest in the understanding of the role of neuroinflammation in the pathophysiology of early brain injury and DCI after SAH. Inflammatory biomarkers are associated with the occurrence of ACV, DCI, and unfavorable outcome. However, the use of anti-inflammatory agents was studied in only a small numbers of subjects. Acetylsalicylic acid, other NSAIDs, thromboxane synthase inhibitors, steroids, nitric oxide donors, and immunosuppressant therapies have not shown beneficial clinical effects. On the other hand, none have been studied in enough detail or in adequate, well-controlled clinical trials to reach a definitive conclusion about safety and efficacy. Currently, there is no intervention directly developed and approved to target neuroinflammation after SAH, therefore anti-inflammatory treatments are not suggested in SAH, at least in the doses and dose regimens studied.

## Author Contributions

AM wrote the first version of the manuscript. RM made crucial extensive revisions.

## Conflict of Interest Statement

RM receives grant support from the Brain Aneurysm Foundation, Canadian Institutes for Health Research, Genome Canada, and the Ontario Genomics Institute; and he is Chief Scientific Officer of Edge Therapeutics, Inc. The other author declares that the research was conducted in the absence of any commercial or financial relationships that could be construed as a potential conflict of interest.
